# Effects of trunk posture on cardiovascular and autonomic nervous systems: A pilot study

**DOI:** 10.3389/fphys.2022.1009806

**Published:** 2022-10-18

**Authors:** Hao Wang, Xiaolin Gao, Yongjin Shi, Dongzhe Wu, Chuangtao Li, Wendi Wang

**Affiliations:** ^1^ Sports Rehabilitation Research Center, China Institute of Sport Science, Beijing, China; ^2^ Department of Sports and Arts, China Agricultural University, Beijing, China

**Keywords:** trunk, posture, autonomic nerve, heart rate variability, cardiac function, spine

## Abstract

**Objective:** Although regular and moderate physical activity has been shown to improve the cardiovascular and autonomic nervous systems, little has been done to study the effects of postural changes in the movement on the heart and autonomic nervous system. To uncover changes in cardiac function and autonomic nerves induced by different underlying posture transitions and explore which trunk postures lead to chronic sympathetic activation. Therefore, this study investigated the effects of trunk posture on the cardiovascular and autonomic nervous systems.

**Methods:** Twelve male subjects (age 24.7 ± 1.3) underwent this study. The non-invasive cardiac output NICOM monitoring equipment and the FIRSTBEAT system are used to dynamically monitor seven trunk postures in the sitting position simultaneously (neutral position, posterior extension, forward flexion, left lateral flexion, right lateral flexion, left rotation, right rotation). Each posture was maintained for 3 min, and the interval between each movement was 3 min to ensure that each index returned to the baseline level. Repeated analysis of variance test was used to compare and analyze the differences in human cardiac function, heart rate variability index, and respiratory rate under different postures.

**Results:** Compared with the related indicators of cardiac output in a neutral trunk position: the cardiac index (CI) was significantly reduced in forwarding flexion and left rotation (3.48 ± 0.34 vs. 3.21 ± 0.50; 3.48 ± 0.34 vs. 3.21 ± 0.46, Δ L/(min/m^2^)) (*p* = 0.016, *p* = 0.013), cardiac output decreased significantly (6.49 ± 0.78 vs. 5.93 ± 0.90; 6.49 ± 0.78 vs. 6.00 ± 0.96, Δ L/min) (*p* = 0.006, *p* = 0.014), the stroke volume (stroke volume)decreased significantly (87.90 ± 15.10 vs. 81.04 ± 16.35; 87.90 ± 15.10 vs. 79.24 ± 16.83, Δ ml/beat) (*p* = 0.017, *p* = 0.0003); heart rate increased significantly in posterior extension (75.08 ± 10.43 vs. 78.42 ± 10.18, Δ beat/min) (*p* = 0.001); left rotation stroke volume index (SVI) decreased significantly (47.28 ± 7.97 vs. 46.14 ± 8.06, Δ ml/m^2^) (*p* = 0.0003); in the analysis of HRV-related indicators, compared with the neutral trunk position, the LF/HF of the posterior extension was significantly increased (1.90 ± 1.38 vs. 3.00 ± 1.17, *p* = 0.037), and the LF/HF of the forward flexion was significantly increased (1.90 ± 1.38 vs. 2.85 ± 1.41, *p* = 0.041), and the frequency-domain index LF/HF of right rotation was significantly increased (1.90 ± 1.38 vs. 4.06 ± 2.19, *p* = 0.008). There was no significant difference in respiratory rate (*p* > 0.05).

**Conclusion:** A neutral trunk is the best resting position, and deviations from a neutral trunk position can affect the cardiovascular and autonomic nervous systems, resulting in decreased stroke volume, increased heart rate, and relative activation of sympathetic tone.

## 1 Introduction

Various diseases caused by poor posture have attracted public attention. And good posture is the regular performance of the human skeleton and muscle tissue. The transformation of various postures can only be carried out under the multiple coordination of human vision, movement, vestibule, and nervous system. Posture training is a method to optimize body shape, stretch ligaments, improve balance, and correct pelvic spine tilt or facet joint disorders caused by poor posture (Fontana Carvalho et al., 2020; Liu et al., 2020; Park et al., 2021; Kerbrat et al., 2022).

Most previous studies on the effects of posture or body position on autonomic function have only involved lying, sitting, standing, and squatting positions (Grant et al., 2012; Abad et al., 2017; Hnatkova et al., 2019; Espinoza-Valdes et al., 2020; Balestrini et al., 2021), and it has been demonstrated that switching from resting to active support results in an increase in heart rate and a decrease in HRV. There are currently no studies on the effects of changes in trunk posture on cardiac function and the autonomic nervous system.

The preganglionic neurons of the cardiac sympathetic nerves run in the lateral horn of the spinal cord T1-5, and their preganglionic fibers originate from the corresponding spinal segment and terminate in the paravertebral ganglia or prevertebral ganglia. The postganglionic fibers are located in the stellate and cervical ganglion within the sympathetic ganglia (Szulczyk and Szulczyk, 1987). Studies have shown that the T1-5 spinal cord segments are associated with sympathetic innervation of the heart, lungs, and upper extremity cardiovascular system, and after massage on the spine, the cardiovascular system, such as blood pressure and heart rate, changes (Mcguiness et al., 1997; Budgell and Polus, 2006; Younes et al., 2017; Amoroso Borges et al., 2018; Picchiottino et al., 2019). Theoretically, changes in the posture of the trunk may affect the tone of the sympathetic nerves on both sides of the spine, leading to changes in the autonomic nervous system and cardiovascular system, which in turn modulate visceral activity or improve chronic stress.

Current research has demonstrated that posture correction, postural training, yoga, Tai Chi, Qigong, and other posture-related exercises or studies can have a good interference effect on chronic diseases (Chan et al., 2019; Gouw et al., 2019; Guo et al., 2020; Oshikawa et al., 2020; Siu et al., 2021). These exercises involve the postural shifts in this study. We designed this study based on the hypothesis that changes in posture affect the cardiovascular and autonomic nervous systems in humans to identify the underlying reasons for improving cardiovascular and autonomic nervous systems. Analysis of changes in cardiac function and autonomic nerves induced by different basic posture transitions and to provide reference and scientific research data reference for the formulation of follow-up related disease prevention and control measures.

## 2 Materials and methods

### 2.1 Participants

Fifteen healthy adult males were recruited. Subjects with cardiovascular and cerebrovascular, metabolic diseases, motor, respiratory, and nervous systems diseases, recent surgery, and history of traumatic pain were excluded. Ultimately 12 subjects were eligible to participate. Before the experiment, the subjects were informed of the basic case, the subjects’ consent was obtained, and informed consent was signed. All subjects fasted for 2 hours before the experimental test and were not allowed to drink caffeinated beverages, alcohol, and other foods and drugs that interfered with the test results within 24 h. This study was reviewed and approved by the Ethics Committee of the Institute of Sports Science of the State Sports General Administration (ethics number: 2022-5-17). G Power 3.1 was used to calculate the sample size before recruiting the subjects. The preliminary experiment shows that the Partial minimum η^2^ = 0.617, and the sample size shows that at least six subjects are needed. General information on subjects is shown in Table 1.

**TABLE 1:** General information of subjects (*n* = 12).

Characteristic	Value
Age (years)	24.7 ± 1.3
Height (cm)	174.3 ± 6.1
Mass (kg)	72.1 ± 10.8
BMI (kg/m^2^)	23.7 ± 3

Values are means ± SD.BMI, body mass index.

### 2.1 Experimental design

The experiment started at 8:00 am and was completed by 11:30am. Twelve subjects were randomly divided into six groups of two. A set of experiments and indicators are tested simultaneously. Experiments were conducted within 1 day, and to avoid disruption of HRV by eating, subjects were instructed to fast for the first 2 hours of the intervention.

**FIGURE 1:**
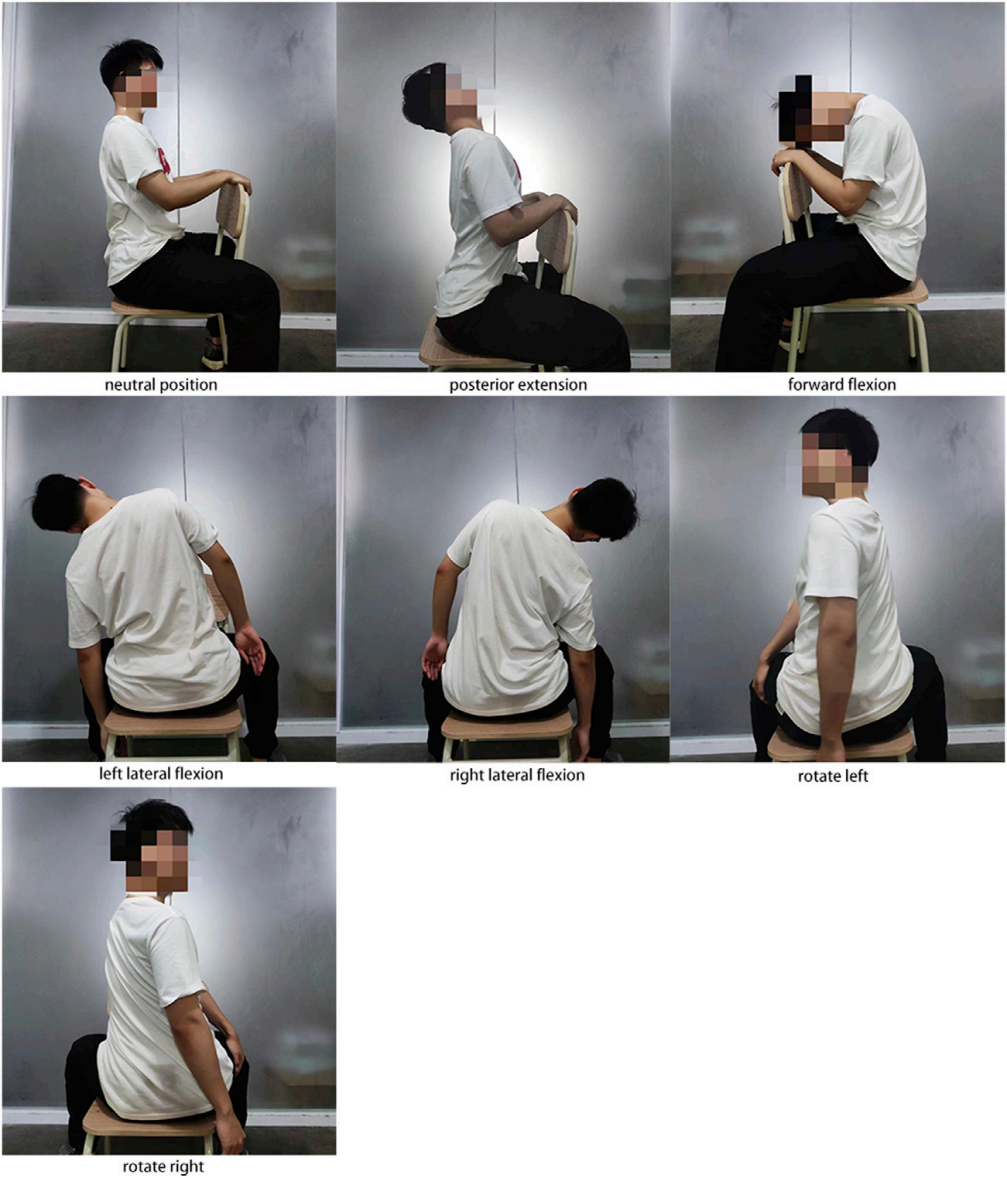
Schematic diagram of seven postures.

Before the experiment, the subjects were taught uniformly to ensure that the candidates mastered all the essentials of movement. All movements are naturally relaxed, avoiding active muscle contractions as much as possible; each pose is about 3 min apart to allow the heart rate to return to a calm state. The subjects held the trunk in a neutral position for 5 min to allow the heart rate to return to a steady state, then performed the following actions in sequence. Although short-term HRV measurements were performed for 5 min in most studies, we found that subjects could not hold certain positions for 5 min, especially extension and lateral flexion. To ensure that active muscle contractions did not affect the design of this study, we set the posture to hold for 3 min. We observed that HRV exhibited similar regular fluctuations at 3 min as measured at 5 min. And the study showed that when HRV was measured in the supine position, there was no significant difference in HRV detected when the cycle was 3 min, and the procedure was 10 min (Grant et al., 2011). As the Figure 1 shows.

1) Neutral trunk posture: The subject’s lower limbs were immobilized, sitting on a chair with legs apart, holding the back of the chair with both hands, and keeping the torso in an upright neutral position for about 3 min.
2) Posterior extension: Under the action of gravity, the head is naturally tilted back, and the trunk is extended back for 3 min.
3) Forward flexion: Under the action of gravity, the head droops naturally; the trunk is flexed forward for 3 min.
4) Left lateral flexion movement: Under the action of gravity, the trunk naturally bends to the left side for 3 min.
5) Right lateral flexion: Under the action of gravity, the trunk naturally bends to the right for 3 min.
6) Left rotation: rotate the torso to the left by 45 degrees, place the right hand on the outside of the left thigh, and hold for 3 min.
7) Right rotation: rotate the trunk 45 degrees to the right, place the left hand on the outside of the right thigh, and hold for 3 min.

### 2.3 Test indicators and tools

#### 2.3.1 Cardiac function indicators

The NICOM Reliant system (Cheetah Medical, United States) was used, which is a portable, non-invasive cardiac output monitoring device based on bio-reactance technology. Subjects were tested for the following hemodynamic indicators: cardiac output (CO), stroke volume (SV), stroke volume variation (SVV), Stroke volume index (SVI), Cardiac index (CI), heart rate (HR), etc.

#### 2.3.2 Heart rate variability index and respiratory rate

The FIRSTBEAT SPORTS system (version 4.7.3.1 Copyfight Firstbeat Technologies Ltd., Jyvaskyla, Finland) was used to collect ECG and analyze HRV data. The data collection time includes 5 min after the resting state, 3 min for each posture transition and maintenance, and 3 min for the recovery period between each posture. The research indicators are time-domain indicators of RR interval and HRV (root mean square value RMSSD of the difference between adjacent NN intervals in the whole process) and frequency-domain indicators (low-frequency LF, high-frequency HF, Low Frequency/High Frequency (LF/HF) ratio).

**FIGURE 2:**
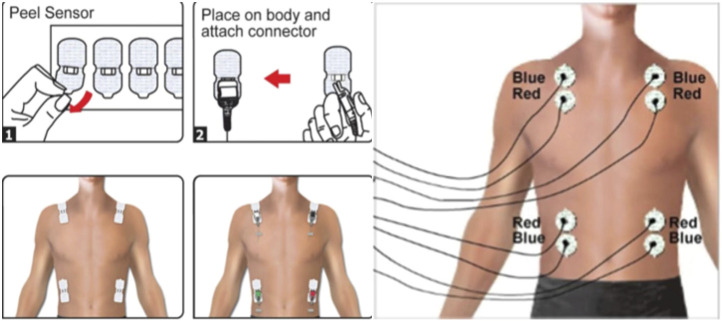
NICOM Reliant system schematic diagram of electrode position.

**FIGURE 3:**
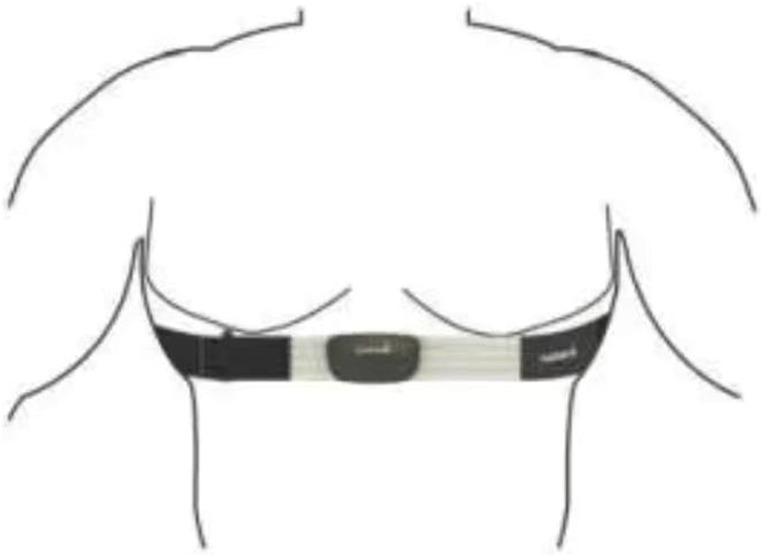
FIRSTBEAT SPORTS system schematic diagram of the binding position of the heart rate belt.

During the experiment, the NICOM monitoring equipment and the FIRSTBEAT system are used to dynamically monitor seven trunk postures in the sitting position simultaneously. NICOM Reliant system and FIRSTBEAT SPORTS system are both non-invasive monitoring devices, and the wearing plan is shown in the Figures 2, 3. The room temperature in the laboratory is 20°C–24°C, and the humidity is 50%–56%.

### 2.4 Statistical analysis

Biostatistics experts from the Institute of Sports Science of the State Sports General Administration reviewed the statistical methods of the article. All values are reported as means ± SD unless stated otherwise, and all statistical analyses were performed using SPSS software (Ver.26; IBM, Armonk, NY). HF/ms^2^ did not conform to the normal distribution by the Shapiro-Wilks test normality test. Using the Kruskal-Wallis test for HF/ms^2^, HF conforms to a normal distribution after logarithmic transformation. Then, one-way repeated measures ANOVA was used to test the cardiac function, heart rate variability, and respiratory rate in seven different postures (neutral, forward flexion, posterior extension, left lateral flexion, right lateral flexion, left rotation, and right rotation), and Graphpad prism 9.0 was used to make graphs. *p* < 0.05 indicated that the difference was statistically significant.

## 3 Results

### 3.1 Cardiac output indicators and heart rate statistics for seven postures

**FIGURE 4:**
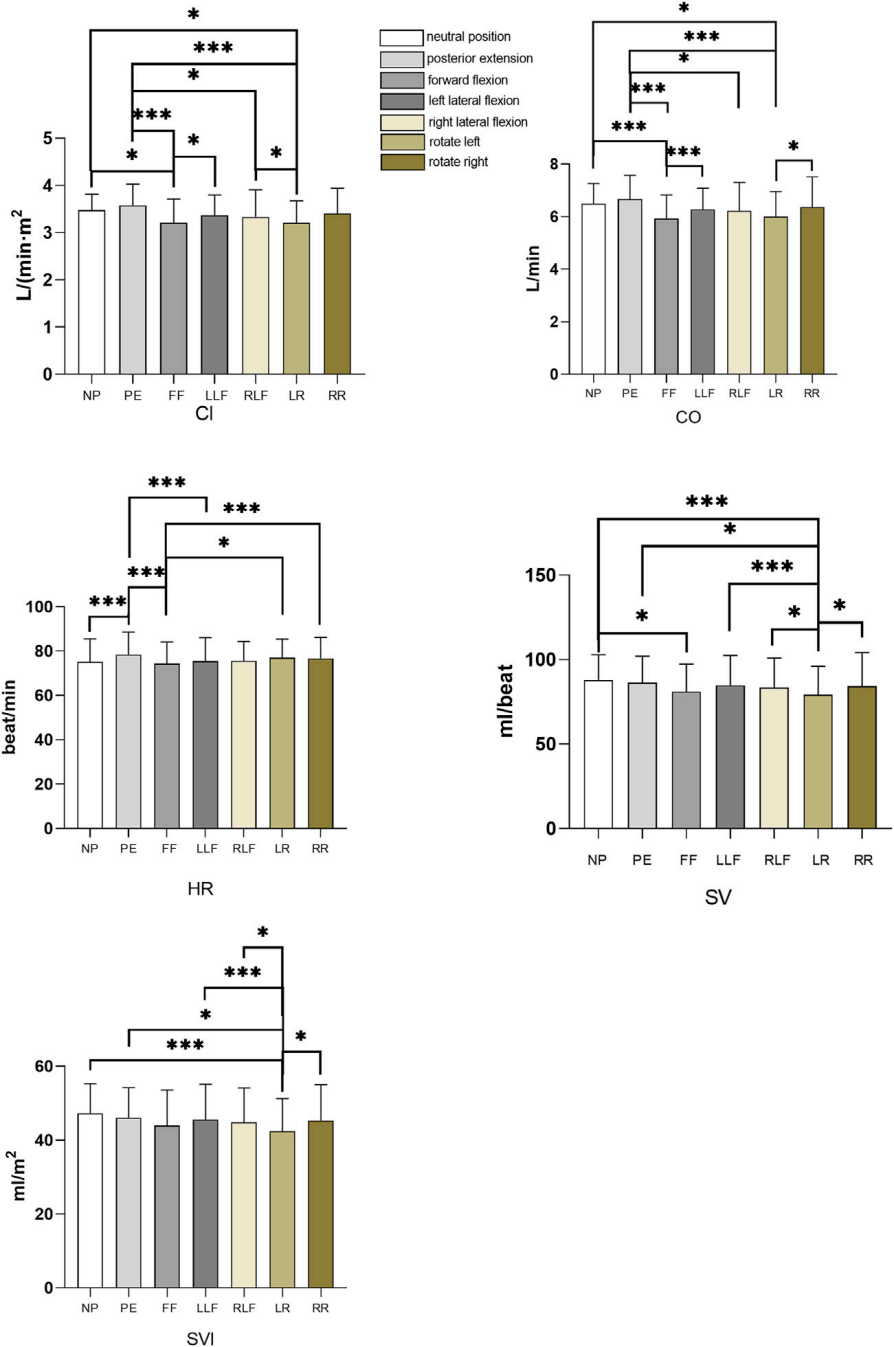
Histogram of cardiac index, cardiac output, heart rate, stroke volume and stroke index of seven postures. NP, neutral position; PE, posterior extension; FF, forward flexion; LLF, left lateral flexion; RLF, right lateral flexion, LR, left rotation, RR, right rotation. *p < 0.05, ***p < 0.01.

CI: The CI of the forward flexion posture was significantly lower than that of the neutral trunk position and the left flexion posture (*p* = 0.016, *p* = 0.046), and the CI of the left rotation posture was significantly lower than that of the neutral trunk position and the left flexion posture (*p* = 0.013, *p* = 0.035), the CI of the posterior extension was significantly higher than that of the forward flexion and left rotation postures (*p* = 0.006, *p* = 0.006); CO: The CO in the forward flexion and left rotation posture was significantly lower than that in the neutral position of the trunk (*p* = 0.006, *p* = 0.014), and the CO in the posterior extension was significantly higher than that in the forward flexion, right flexion, and left rotation posture (*p* = 0.002, *p* = 0.032, *p* = 0.006), the left rotation attitude CO was significantly lower than that of the right rotation (*p* = 0.045); HR: HR in posterior extension was significantly higher than that in a neutral position, forward flexion, and left flexion (*p* = 0.001, *p* = 0.005, *p* = 0.008), and HR in forwarding flexion was significantly lower than that in left rotation, right rotation HR (*p* = 0.02, *p* = 0.007); SV: The SV of the left rotation posture was significantly lower than that of the neutral trunk, posterior extension, right flexion, and right rotation posture (*p* = 0.0003, *p* = 0.014, *p* = 0.007, *p* = 0.024, *p* = 0.031); left flexion posture SV Significantly higher than left rotation (*p* = 0.024); The SV in forwarding flexion is significantly lower than in the neutral trunk (*p* = 0.017); SVI: The SVI in the left rotation posture was significantly lower than that in the neutral trunk, posterior extension, left flexion, right flexion, and right rotation posture (*p* = 0.0003, *p* = 0.015, *p* = 0.007, *p* = 0.031, *p* = 0.021). As shown in Table 2 and Figure 4.

**TABLE 2:** Effects of different sitting postures on cardiac output and heart rate (*n* = 12).

Index	NP	PE	FF	LLF	RLF	LR	RR
CI (L/(min·m^2^))	3.48 ± 0.34^e^ ^,^ ^k^	3.58 ± 0.45^f^ ^,^ ^i^ ^,^ ^l^	3.21 ± 0.50^a^ ^,^ ^d^ ^,^ ^g^	3.36 ± 0.43^e^ ^,^ ^k^	3.33 ± 0.58^c^	3.21 ± 0.46^a^ ^,^ ^d^ ^,^ ^i^	3.41 ± 0.53
CO (L/min)	6.49 ± 0.78^f^ ^,^ ^k^	6.67 ± 0.91^f^ ^,^ ^i^ ^,^ ^l^	5.93 ± 0.90^b^ ^,^ ^d^ ^,^ ^h^	6.28 ± 0.81^f^	6.22 ± 1.09^c^	6.00 ± 0.96^a^ ^,^ ^d^ ^,^ ^m^	6.37 ± 1.15^l^
HR (beat/min)	75.08 ± 10.43^d^	78.42 ± 10.18^b^ ^,^ ^f^ ^,^ ^h^	74.33 ± 9.79B^k^ ^,^ ^n^	75.42 ± 10.64^d^	75.50 ± 8.89	76.92 ± 8.57^e^	76.67 ± 9.56^f^
SV (ml/beat)	87.90 ± 15.10^e^ ^,^ ^l^	86.30 ± 15.71^k^	81.04 ± 16.35^a^	84.87 ± 17.42^l^	83.55 ± 17.45^k^	79.24 ± 16.83^b^ ^,^ ^c^ ^,^ ^h^ ^,^ ^i^ ^,^ ^m^	84.49 ± 19.65^k^
SVI (ml/m^2^)	47.28 ± 7.97^l^	46.14 ± 8.06^k^	43.92 ± 9.64	45.53 ± 9.63^l^	44.83 ± 9.30^k^	42.44 ± 8.81^b^ ^,^ ^c^ ^,^ ^h^ ^,^ ^i^ ^,^ ^m^	45.30 ± 9.74^k^

Data are expressed as mean ± SD. NP, neutral position; PE, posterior extension; FF, forward flexion; LLF, left lateral flexion; RLF, right lateral flexion; LR, left rotation; RR, right rotation.

^a^ *p* < 0.05 current posture vs. neutral position of the trunk posture

^b^ *p* < 0.01 current posture vs. neutral position of the trunk posture

^c^ *p* < 0.05 current posture vs. posterior extension position

^d^ *p* < 0.01 current posture vs. posterior extension position

^e^ *p* < 0.05 current posture vs. forward flexion position

^f^ *p* < 0.01 current posture vs. forward flexion position

^g^ *p* < 0.05 current posture vs left lateral flexion position

^h^ *p* < 0.01 current posture vs. left lateral flexion position

^i^ *p* < 0.05 current posture vs. right lateral flexion position

^j^ *p* < 0.01 current posture vs. right lateral flexion position

^k^ *p* < 0.05 current posture vs. left rotation position

l *p* < 0.01 current posture vs. left rotation position

^m^ *p* < 0.05 current posture vs. right rotation position

^n^ *p* < 0.01 current posture vs. right rotation position

### 3.2 Seven posture heart rate variability statistics

**FIGURE 5:**
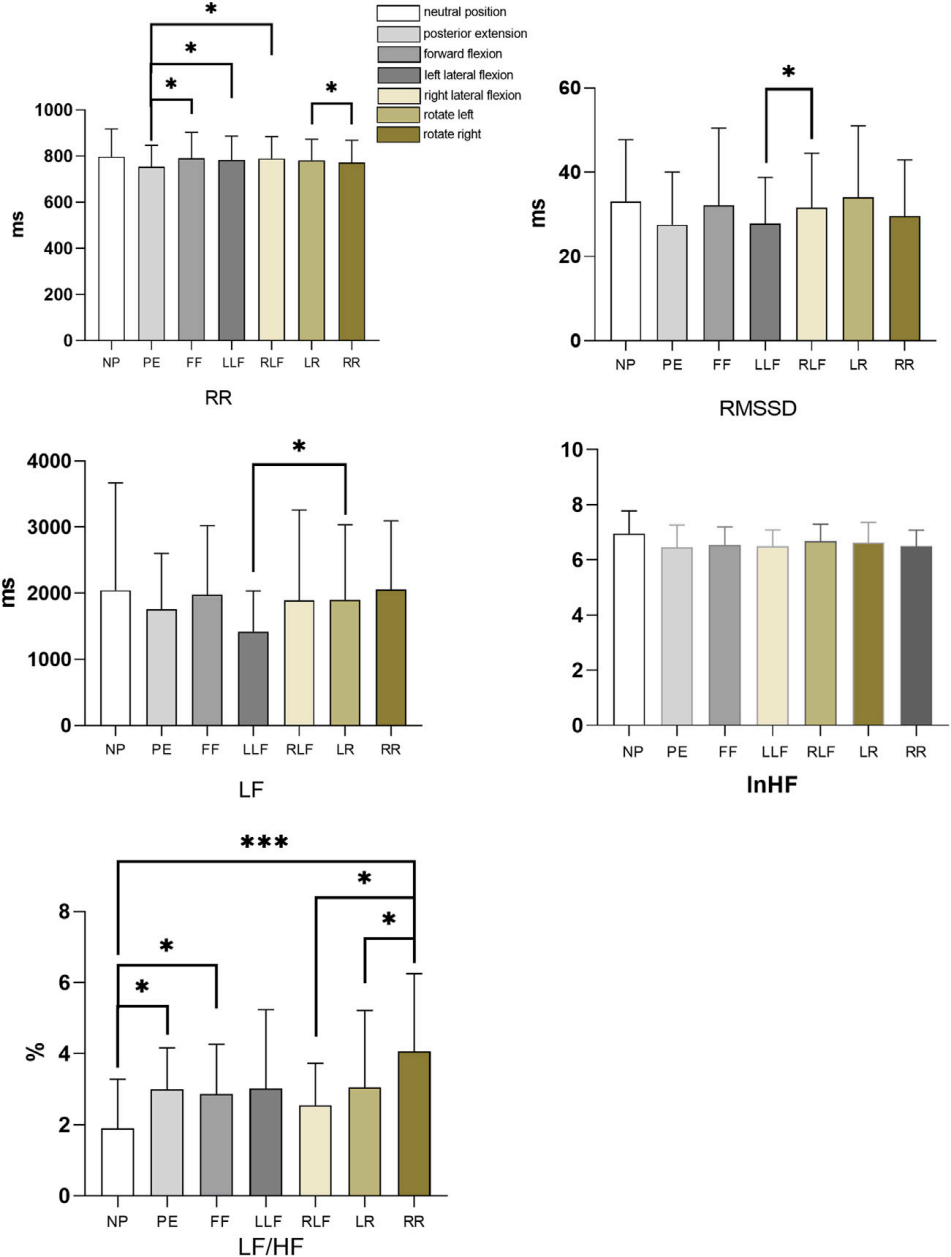
Histogram of heart rate variability (RR, RMSSD, LF, lnHF, LF/HF) for seven postures. NP stands for neutral, PE stands for posterior extension, FF stands for forward flexion, LLF stands for left lateral flexion, RLF stands for right lateral flexion, LR stands for left rotation, RR stands for right rotation. *p < 0.05, ***p < 0.01.

RR: The RR of the posterior extension was significantly lower than that of forwarding flexion, left flexion, and right flexion posture (*p* = 0.047, *p* = 0.026, *p* = 0.025), and the RR of right flexion posture was significantly higher than that of right rotation posture (*p* = 0.020); RMSSD: The RMSSD of the left flexion posture was significantly lower than that of the right flexion posture (*p* = 0.031); LF: The left flexion posture LF was significantly lower than the left rotation posture (*p* = 0.05); LF/HF: The LF/HF in the neutral position of the trunk was significantly lower than that in the posterior extension, forward flexion, and right rotation postures (*p* = 0.037, *p* = 0.041, *p* = 0.008), and the LF/HF in the right rotation posture was significantly higher than that in the right flexion posture (*p* = 0.004), the right rotation LF/HF was significantly higher than the left rotation attitude (*p* = 0.044). Respiratory rate statistics for seven postures. As shown in Table 3 and Figure 5. And HF Kruskal-Wallis H test as shown in Table 4.

**TABLE 3:** Effects of different sitting postures on heart rate variability (*n* = 12).

Index	NP	PE	FF	LLF	RLF	LR	RR
RR (ms)	797.24 ± 120.79	753.81 ± 93.6^e^ ^,^ ^g^ ^,^ ^i^	790.52 ± 112.33^c^	783.08 ± 103.78^c^	789.75 ± 95.24^c^ ^,^ ^m^	780.85 ± 92.05	772.04 ± 96.64^i^
RMSSD (ms)	33 ± 14.75	27.5 ± 12.54	32.17 ± 18.31	27.83 ± 10.94^i^	31.58 ± 12.94^g^	31.75 ± 11.63	29.58 ± 13.34
lnHF (ms^2^)	6.95 ± 0.84	6.45 ± 0.81	6.54 ± 0.66	6.49 ± 0.59	6.68 ± 0.62	6.63 ± 0.74	6.49 ± 0.60
LF (ms^2^)	2044.87 ± 1622.97	1757.05 ± 844.45	1974.01 ± 1046.59	1417.19 ± 619.1^l^	1891.11 ± 1368.34	1903.32 ± 1133.19^z^	2055.71 ± 1040.37
LF/HF (%)	1.90 ± 1.38^c^ ^,^ ^e^ ^,^ ^n^	3.00 ± 1.17^a^	2.85 ± 1.41^a^	3.03 ± 2.21	2.55 ± 1.18^n^	3.05 ± 2.17^m^	4.06 ± 2.19^b^ ^,^ ^j^ ^,^ ^k^

Data are expressed as mean ± SD. NP, neutral position; PE, posterior extension; FF, forward flexion; LLF, left lateral flexion; RLF, right lateral flexion; LR, left rotation; RR, right rotation

Note: Between poses with statistical significance in Tables 2, 3:

^a^ *p* < 0.05 current posture vs. neutral position of the trunk posture

^b^ *p* < 0.01 current posture vs. neutral position of the trunk posture

^c^ *p* < 0.05 current posture vs. posterior extension position

^d^ *p* < 0.01 current posture vs. posterior extension position

^e^ *p* < 0.05 current posture vs. forward flexion position

^f^ *p* < 0.01 current posture vs. forward flexion position

^g^ *p* < 0.05 current posture vs. left lateral flexion position

^h^ *p* < 0.01 current posture vs. left lateral flexion position

^i^ *p* < 0.05 current posture vs. right lateral flexion position

^j^ *p* < 0.01 current posture vs. right lateral flexion position

^k^ *p* < 0.05 current posture vs. left rotation position

^l^ *p* < 0.01 current posture vs. left rotation position

^m^ *p* < 0.05 current posture vs. right rotation position

^n^ *p* < 0.01 current posture vs. right rotation position

**TABLE 4:** HF Kruskal-Wallis H test.

position	HF median (P25, P75)
neutral position	1329.56 (497.66,1606.97)
posterior extension	808.43 (372.46,1093.24)
left lateral flexion	636.16 (411.15,1392.90)
right lateral flexion	726.17 (488.05,972.85)
rotate left	640.14 (499.01,1139.10)
rotate right	753.66 (404.60,1053.55)
Z	3.76
P	0.709

Data are expressed as median (P25, P75). NP, neutral position; PE, posterior extension; FF, forward flexion; LLF, left lateral flexion; RLF, right lateral flexion; LR, left rotation; RR, right rotation.

## 4 Discussion

Former studies have found that cardiac output-related indicators are closely related to various factors. Among them, the gravitational factor is a key factor in cardiac preload and myocardial contractility. Changes in body position can cause cardiovascular reflexes, causing orthostatic venous return obstruction, which leads to reduced cardiac output (Bergenwald et al., 1977; Matzen et al., 1991; Harms et al., 2003; Dorogovtsev et al., 2021; Whittle et al., 2022). During supine head-up tilt (HUT), a reduction in SV is associated with a drop in pleural fluid content, cardiac volume, central blood pressure, and hemodynamic changes (Van Lieshout et al., 2005; Cheung et al., 2020; Dorogovtsev et al., 2021). At the same time, cardiac output is also affected by heart rate and left ventricular stroke volume. The factors determining SV include cardiac preload, afterload, myocardial contractility, and ventricular compliance (Luecke and Pelosi, 2005). Due to the influence of the respiratory pump, the volume of the thoracic cavity increases during inspiration, and the negative pressure in the pleural cavity increases during inspiration, increasing venous return to the heart and an increase in the preload of the heart, which leads to an increase in SV. Conversely, when the volume of the thoracic cavity decreases, the venous return to the heart decreases, and the decrease in cardiac preload leads to reduced SV. And some studies have found that with the increase of inspiratory depth, the volume of the thoracic cavity increases, and the volume of venous return to the heart increases with the increase of inspiratory depth (Stolz et al., 2009). After comparing five different inclination positions (sitting without back, 80°, 65°, 40° back, and passive), it was found that a gradual increase in trunk inclination results in a gradual decrease in thoracic displacement and tidal volume (Romei et al., 2010).

This study found that compared with the SV in the neutral trunk position, the SV of the other six postures decreased to varying degrees. The reason may be that the six postures caused the reduction of the thoracic volume, which reduced the left ventricular end-diastolic blood filling volume and decreased preload. At the same time, the increase of intrathoracic pressure leads to the rise of cardiac afterload, and the decrease of cardiac preload and the growth of afterload work together to cause the SV to decrease. The SV decreased most significantly in forwarding flexion and left rotation (*p* < 0.05). The significant decrease in SV in forwarding flexion may be that the thoracic cage is more compressed in this posture. In addition to the above reasons, the significant decrease in SV during left rotation may be related to the asymmetry of human anatomical structure and function. The heart is located between the two lungs above the diaphragm in the thoracic cavity, to the left of the midline. The right lung has three lobes, which are wide and short, and the left lung has two lobes, which are narrow and long. During left rotation, the right thorax is in the expiratory position, the left thorax is in the inhalation position, and the right lung is squeezed. Since the volume of the right lung is larger than that of the left lung, the increase in pulmonary circulation resistance during left rotation is greater than that in the right rotation posture. The central venous pressure was larger during left rotation, which affected the venous return to the heart, resulting in a statistically significant difference between left and right rotation SV. It may be because the thoracic rotation exerts more compression on the thorax and lungs than the lateral and forward flexion postures, ultimately resulting in the smallest SV in the left-rotated posture.

The interaction of sympathetic and parasympathetic nerves results in slight differences in the RR interval of consecutive heartbeats, thereby giving rise to variability in the cardiovascular system known as heart rate variability (Xhyheri et al., 2012; Schiweck et al., 2019). Some diseases or other reasons cause changes in the balance of the cardiac sympathetic and vagus nerves, which can lead to changes in heart rate (HR), HRV, and cardiovascular system dysfunction. (Benichou et al., 2018; Hayano and Yuda, 2019; Fournié et al., 2021). The commonly used analysis methods of HRV mainly include frequency-domain analysis and time-domain analysis methods. The indicators corresponding to the frequency domain analysis method include LF, HF, and LF/HF. Sympathetic and parasympathetic nerves jointly regulate LF, and parasympathetic nerves mainly control HF. LF/HF represents the balance between sympathetic nerves and parasympathetic nerves. An increase in the ratio indicates sympathetic activity is predominant, whereas a decrease in this ratio means parasympathetic activity is predominant. The corresponding indicators of the time-domain analysis method include RMSSD, SDNN, and PNN50, of which the time-domain indicators RMSSD and pNN50 and the frequency-domain indicator HF represent parasympathetic nervous tension, SDNN reflects the overall situation of HRV (Electrophysiology TFESCNASP, 1996).

Previous studies have found that changing posture can cause changes in HRV. After studying the HRV of three sleeping positions: supine, right lateral, and left lateral position, it was found that the HF in the right lateral place was higher than in other sleeping positions and the lower LF/HF ratio, suggesting that the vagus nerve regulation in the right lateral position more capable (Kuo and Chen, 1998). HRV in the standing position is lower than that in the supine position, and heart rate is higher than that in the supine position, indicating that the standing position is higher than the supine position in cardiac autonomic pressure (Grant et al., 2012; Abad et al., 2017; Vescovi, 2019). At the same time, some studies also found that the increase in heart rate after changing from the supine position to the unsupported sitting position is not higher than that of the standing position, indicating that the sympathetic nervous control is enhanced in the supine, sitting, and standing positions in turn (Hnatkova et al., 2019).

To date, no relevant studies have emerged regarding the effect of different trunk postures on HRV. This study found that RMSSD, HF, and ln HF, representing vagal activity, decreased in six postures other than the neutral trunk, while LF/HF, an indicator of autonomic balance, increased. The index LF, representing co-regulation of sympathetic and vagus nerves, fluctuates slightly. Changes in body position have been found to affect the resistance or compliance of the lungs and thorax, affecting respiratory volume and frequency (Stewart et al., 2018; Mendes et al., 2020; Sharma et al., 2020). Meanwhile, studies have shown that the frequency domain indicators of HRV are affected by changes in breathing patterns (Sanderson et al., 1996; Bernardi et al., 2000; Lin et al., 2020; Shaffer and Meehan, 2020). Although the seven different postures in this study had different effects on thoracic or lung volume, repeated measures ANOVA found no significant difference in respiratory rate among the seven postures, As shown in Table 5, indicating that the respiratory rate in this study had little effect on HRV.

**TABLE 5:** Effects of different sitting postures on respiratory rate (n = 12).

Posture	NP	PE	FF	LLF	RLF	LR	RR
Breath rate (beat/min)	15.24 ± 3.27	14.8 ± 2.43	14.77 ± 3.49	15.73 ± 2.6	15.73 ± 2.66	16.80 ± 3.09	15.60 ± 3.15

Values are means ± SD. NP, neutral position; PE, posterior extension; FF, forward flexion; LLF, left lateral flexion; RLF, right lateral flexion; LR, left rotation; RR, right rotation.

Regarding the study of stimulating the spine or other stress on the spine affecting the activity of the autonomic nerve, and found that the manipulation of the thoracic spine significantly increased the LF, LFnorm, and LF/HF indexes of the HRV of the subjects, indicating that the manipulation of the thoracic spine improved the activity of the sympathetic nerve (Budgell and Polus, 2006). Clinical evidence shows that patients with adolescent idiopathic scoliosis (AIS) had significantly higher overall SNS activity than normal subjects (Enslein and Chan, 1987). Scoliosis is more common in the thoracic and thoracolumbar segments, which may be related to the fact that the ganglia of the sympathetic nervous system are located in front of the thoracic costal vertebral joints. SNS activity was increased in healthy subjects but not in paraplegic and tetraplegic patients during head-up tilt (HUT) supine position since the majority of peripheral sympathetic nerves in paraplegic and tetraplegic patients are separated from the control of the spine. After spinal cord injury, the sympathetic nerves lose most of the power of the spinal cord, resulting in the curvature of the spine being unable to alter sympathetic nerve activity significantly (Houtman et al., 2000; Wecht and Bauman, 2018). They all indicate that the course of sympathetic nerves in the spine is significant to cardiovascular function and the autonomic nervous system.

Former studies have found that HR, mean arterial pressure, and left ventricular ejection time vary with posture (Olschewski and Brück, 1990). Heart rate and myocardial contractility are both affected by autonomic nerves. This study found that heart rate changes to varying degrees in different postures, especially in the posterior extension. In HRV analysis, the frequency-domain indices LF/HF were significantly increased in posterior extension, forward flexion, and right rotation relative to the neutral trunk. From the analysis of the mean changes, HF and LF both decreased during extension and forward flexion, and the decrease in HF was greater than the decrease in LF. During right rotation, LF slightly increased, and HF decreased. It indicates that the posture of stretch back, forward flexion, and right rotation will decrease parasympathetic nerve activity and a relative increase in sympathetic nerve activity. Anatomically, the sympathetic nervous system ganglia are located in front of the thoracic vertebral joints. From a biomechanical perspective, the extension and flexion of the spine stretch the sympathetic nerves, which in turn increases sympathetic nerve activity. This view in this study seems to explain why people feel refreshed after doing a stretch or neck stretch after sitting for a long time. This is because the movement of the spine in the sagittal axis activates the sympathetic nerves.

After excluding the two special postures of flexion and extension (sagittal spine motion posture), the analysis found that the HF decreased relatively more when the left flexion or right rotation postures were maintained. In the left flexion and right rotation postures, the left thorax is in the expiratory position, and the right thorax is in the inspiratory position. This suggests that the left thorax in the expiratory position may be associated with the decreased parasympathetic activity. This is consistent with the results of previous studies. Normal sleeping position found that HF was the lowest and LF/HF was the highest when lying on the left side (Kuo and Chen, 1998). The sleeping position on the left side is similar to the left lateral flexion and right rotation of the thorax in this experiment. That is, both are affected by the left thorax. During compression, the left thorax is in the expiratory position. This finding will be followed up by increasing the sample size of the subjects when the as experimental conditions permit.

The frequency-domain index LF/HF analysis showed that the sympathetic and parasympathetic tensions were relatively balanced at rest, and other postures showed sympathetic dominance (high LF/HF), suggesting that the neutral posture of the trunk is the best resting posture. Flexion and extension, lateral flexion, and rotation may stimulate sympathetic activation. People sometimes rest on the table in a forward-bent position. Although this posture can relieve muscle fatigue, it still stimulates the sympathetic nerves continuously and does not achieve real rest. Force contraction will reflexively activate the sympathetic nerve, and increase HR and blood pressure. This study was performed in a sitting position, during which each posture was a relaxed state of inactive muscle contractions, intending to minimize the effects of lower limbs and muscle contractions on the autonomic and cardiovascular systems (Smith et al., 2006; Wang et al., 2010; Kim et al., 2020; Lee et al., 2021).

## 5 Conclusion

In conclusion, this study found that trunk forward flexion, posterior extension, lateral flexion, and trunk rotation can lead to changes in heart rate, cardiac function, and autonomic nerves. The range of the changes is closely related to different postures. Specifically, the heart rate was significantly increased in the posterior extension posture, the stroke volume was significantly decreased in the left flexion posture, and the LF/HF was increased considerably in the forward flexion, posterior extension, and right rotation posture. The reasons are closely related to cardiac volume, preload and post load, lung anatomy, and sympathetic nerve course on the spine. Keeping the trunk in a neutral position is the best resting position. Deviating from the neutral position of the trunk will affect the cardiovascular and autonomic nervous systems, reduce SV, speed up HR, and relatively increase sympathetic tone.

## Data Availability

The raw data supporting the conclusions of this article will be made available by the authors, without undue reservation.
